# From the Clinic, to the Clinic: Improving the Fluorescent Imaging Quality of ICG via Amphiphilic NIR-IIa AIE Probe

**DOI:** 10.3390/bios16020090

**Published:** 2026-02-01

**Authors:** Anjun Zhu, Zhibo Xiao, Aihui Sun, Feng Lu, Haozhou Tang, Xuekun Zhang, Ran Ren, Wei Yu, Andong Shao, Ninghan Feng, Shouyu Wang, Jianming Ni, Yaxi Li

**Affiliations:** 1 Department of Radiology, Jiangnan University Medical Center, Wuxi No. 2 People’s Hospital, Wuxi 214002, China; 2 Department of Radiology, Wuxi 9th People’s Hospital Affiliated to Soochow University, Wuxi 214062, China; 3 OptiX+ Laboratory, School of Electronics and Information Engineering, Wuxi University, Wuxi 214105, China; 4 Computational Optics Laboratory, School of Sciences, Jiangnan University, Wuxi 214122, China; 5 School of Life Sciences and Health Engineering, Jiangnan University, Wuxi 214122, China; 6 Department of Urology, Wuxi No. 2 People’s Hospital, Wuxi 214002, China

**Keywords:** amphiphilic probe, aggregation-induced emission, second near-infrared imaging, deep learning

## Abstract

Fluorescence imaging is crucial for providing detailed information in clinical practice. However, traditional first near-infrared (NIR-I) dyes such as indocyanine green (ICG) exhibit limitations such as shallow penetration depth, low contrast, and suboptimal clarity due to light scattering and autofluorescence. To overcome these drawbacks, we utilized a novel amphiphilic second near-infrared (NIR-II) aggregation-induced emission (AIE) probe (TCP) with an emission range beyond 1300 nm (NIR-IIa). Using approximately 200 co-registered NIR-I/NIR-IIa image pairs acquired with TCP, we trained a SwinUnet-based deep learning model to transform low-quality NIR-I ICG images into high-resolution NIR-IIa-like images. Owing to its superior brightness and photostability, TCP enhances in vivo fluorescent angiography, offering clearer vascular details and a higher signal-to-background ratio (SBR) in the NIR-IIa region, 2.6-fold higher than that of ICG in the NIR-I region. The deep learning model successfully converted blurred NIR-I images into high-SBR NIR-IIa-like images, achieving rapid imaging speeds without compromising quality. This work introduces a synergistic “probe-plus-AI” paradigm that substantially improves both the quality and speed of clinical fluorescence imaging, providing a pathway that is immediately translatable to enhanced diagnostics and image-guided surgery.

## 1. Introduction

Fluorescence imaging is integral to clinical practice, providing distinguishable and detailed information on lesions [1,2]. First near-infrared (NIR-I, 700–1000 nm) fluorescence imaging, most commonly conducted using indocyanine green (ICG), is used to assist tumor imaging and image-guided surgery [3,4,5,6]. However, it presents several drawbacks due to light scattering and autofluorescence—shallow imaging depth, low contrast, and poor clarity—and can no longer meet the demands of precision medicine. To enhance the performance of clinical fluorescence imaging techniques, researchers have explored strategies such as probe modification, equipment upgrades, and software development [7,8]. Recently, fluorescence imaging in the second near-infrared window (NIR-II, 1000–1700 nm) has been investigated for use as a noninvasive in vivo technique, providing benefits such as deeper tissue penetration, lower background noise, and higher spatial resolution, attributable to reduced light scattering and diminished autofluorescence [9,10,11]. Notably, NIR-II imaging at wavelengths beyond 1300 nm (NIR-IIa) can achieve single-cell resolution at sub-centimeter tissue penetration depths, as demonstrated using novel NIR-IIa probes such as inorganic quantum dots, rare earth-doped nanoprobes, and organic probes [12,13,14,15,16]. However, these novel NIR-IIa probes require a long development cycle, meaning they cannot be prepared for clinical application quickly; additionally, mere equipment upgrades cannot overcome the inherent limitations of optical imaging.

The rapid development of artificial intelligence (AI), particularly deep learning, has created new opportunities to enhance traditional fluorescent imaging modalities [17]. Beyond conventional image processing, deep learning enables powerful computational techniques such as super-resolution reconstruction and noise suppression, which can significantly improve imaging contrast [18,19]. For instance, a deep generative adversarial network (GAN), a type of deep learning algorithm, was used to transform NIR-I fluorescent images into NIR-II forms [20]. Most previous studies on this topic relied on either unpaired public or small-sample datasets; it is worth noting that paired datasets generally result in superior and accurate enhancement due to their inherent pixel-to-pixel correspondence [21,22]. However, acquiring such paired datasets is technically demanding, requiring a fluorophore capable of broad emissions across both the NIR-I and NIR-II regions, along with synchronized spectral acquisition hardware and a sufficient image volume. As a result, deep learning approaches that leverage paired NIR-I and NIR-II datasets remain largely unexplored; it is therefore essential to ensure that the probes are suitable and that there is a sufficient volume of data to enable the training of paired datasets.

NIR-II probes with aggregation-induced emission (AIE) characteristics can exhibit enhanced fluorescence without spontaneous molecular π-π stacking, which triggers aggregation-caused quenching (ACQ) of ICG [23]. Furthermore, NIR-II AIE probes exhibit broad emission and a large Stokes shift, enabling simultaneous NIR-I and NIR-II emission while avoiding interference on the imaging background from the excitation light source [24,25]. In our previous work, we developed a series of NIR-II AIE probes and collected fluorescent images at different wavelengths, ranging from NIR-I to NIR-II [26,27]. In this study, we designed an amphiphilic NIR-II AIE probe (TCP) with a maximum emission of 1080 nm and an emission tail extending beyond 1300 nm and used the SwinUnet framework to train the paired NIR images we collected within the 900–1300 nm range. This approach enables the conversion of blurred NIR images into high signal-to-background ratio (SBR) images in the NIR-IIa region. Leveraging the rapid exposure time characteristic of the NIR-I region, deep learning allowed us to quickly acquire high-resolution, high-SBR NIR-IIa images for noninvasive in vivo vascular imaging. Additionally, we trained artificial neural networks to transform ICG fluorescence images from the NIR-I region into images resembling those in the NIR-IIa region. This work demonstrates an innovative approach that combines NIR-IIa AIE probes with deep learning to convert blurred NIR-I ICG images into much clearer NIR-IIa images that can be obtained more quickly, producing images that closely resemble the ground truth. This presents potential advancements in basic biomedical research and enhancements in clinical diagnostics and image-guided surgery.

## 2. Materials and Methods

### 2.1. Materials and Instruments

ICG was purchased from Beijing Warwick Chemical (Beijing, China). Other reagents were commercially obtained and used without further purification. Absorption spectra were recorded using PUMADA (Shanghai, China) P7 instrument. Emission spectra were recorded on Horiba (Kyoto, Japan) Fluorolog-QM fluorescence spectrophotometer. The average particle sizes were determined by Malvern (Worcestershire, UK) Zetasizer Nano ZSat room temperature. The morphology of particles was observed under JEOL (Kyoto, Japan) JEM-200 transmission electron microscope (TEM). Cell viability assays were conducted on Molecular Devices (Sunnyvale, CA, USA) SpectraMax 190 microplate reader. Blood indicator analysis was recorded by Zybio (Chongqing, China) EXC-400 and Mindray (Shenzhen, China) BC-5000 Vet. Slice sectional images were collected by 3DHistech (Budapest, Hungary) Pannoramic MIDI system. All NIR-II fluorescence images were acquired using an Artemis Intelligent Imaging (Shanghai, China) Mars NIR-II in vivo macro imaging system.

### 2.2. Synthesis and Characterization of TCP

The synthesis of TC was conducted according to a literature procedure and our previous report [27,28]. Detailed information is provided in the supplementary material. Briefly, the precursor compound TCM (120 mg, 0.07 mmol) was dissolved in dichloromethane (DCM, 5 mL) in a 25 mL round-bottom flask. Trifluoroacetic acid (TFA, 1 mL) was then added dropwise to the mixture in an ice bath. The reaction was stirred at room temperature for 6 h. The reaction was monitored by TLC, and upon completion, the solvent was removed under reduced pressure using IKA (Guangzhou, China) rotary evaporator. The crude product was washed with acetonitrile and methanol to afford TC as a dark green solid (90 mg, 94% yield). ^1^H NMR (400 MHz, DMSO-*d*_6_): δ (ppm) 12.13 (s, 4H), 7.62 (d, *J* = 7.8 Hz, 4H), 7.47 (s, 2H), 7.21–7.20 (m, 8H), 6.99–6.95 (m, 12H), 2.80 (s, 8H), 2.54–2.50 (m, 8H), 1.23 (s, 6H), 0.92–0.71 (m, 16H), 0.51–0.36 (m, 12H). ^13^C NMR (101 MHz, CDCl_3_): δ (ppm) 174.31, 173.20, 152.83, 147.80, 145.53, 145.36, 145.27, 144.65, 136.61, 129.95, 128.78, 127.15, 126.80, 126.08, 125.01, 122.48, 115.76, 51.80, 32.15, 30.20, 30.11, 28.17, 25.51, 22.64, 14.23, 10.74.

For the synthesis of TCP, a mixture of TC (6.7 mg, 5 μmol) and HBTU (19 mg, 50 μmol) in N,N-dimethylformamide (DMF, 5 mL) was stirred in a 25 mL round-bottom flask for 30 min. Subsequently, PEG_22_-NH_2_ (50 mg) and DIPEA (20 μL) were added to the mixture. The reaction was allowed to proceed at room temperature for 48 h. Upon completion, the resulting mixture was dialyzed against deionized (DI) water using a dialysis tube (MWCO 3500) for 48 h, with the water being changed 6 times, to remove excess reagents and solvent. After lyophilization, the final product TCP was obtained as a dark green powder. MALDI-TOF-MS: *m*/*z* = 5636.0566.

### 2.3. Characterization of Optical Properties

Stock solutions of ICG and TCP were prepared in pure water at a concentration of 50 mM. For measurements, working solutions were diluted to 10 μM. Absorption and emission spectra (excitation at 808 nm) were recorded. Emission stability was monitored during continuous exposure to an 808 nm laser at a power density of 1 W/cm^2^.

### 2.4. Molecular Dynamics (MD) Simulations

The molecular structure of TCP was constructed using ChemBioDraw 22.0, and its topology file for molecular dynamics simulations was generated with PolyParGen v2. All simulations were performed using Gromacs 2022.4 with the OPLS-AA force field and the TIP3P water model. Ten TCP molecules were placed in a cubic box of 12 nm × 12 nm × 12 nm, with sodium and chloride ions added to achieve a salt concentration of 0.15 mol/L. The system energy was minimized in two steps: first, 10,000 iterations using the steepest descent method, followed by 5000 iterations using the conjugate gradient algorithm. Subsequently, the system was equilibrated with 500 ps of NVT simulation and 500 ps of NPT simulation. A final production simulation was run for 150 ns. The V-rescale and Parrinello-Rahman algorithms were employed for 298.15 K and 1 atmosphere pressure control with a time step of 2 fs. Electrostatic interactions were treated using the Particle Mesh Ewald (PME) method, while van der Waals and Coulomb interactions were truncated at 12 Å. Hydrogen bonds were constrained using the LINCS algorithm. Trajectory analysis was conducted using built-in Gromacs 2022.4 commands, and visualization along with interaction analysis was performed with PyMOL 2.5.0.

### 2.5. Cytotoxicity Assay

The cytotoxicity of TCP was evaluated against 293 cells using Solarbio (Beijing, China) Cell Viability Kit with the 3-(4,5-dimethylthiazol-2-yl)-2,5-diphenyltetrazolium bromide (MTT) assay. Cells were seeded in 96-well plates at a density of 1 × 10^4^ cells per well and incubated for 12 h. Subsequently, the cells were treated with the TCP at concentrations of 0, 10, 25, and 50 μM for 24 h. MTT reagent was then administered according to the manufacturer’s instructions. After incubation, the absorbance at 490 nm was measured using Molecular Devices (Sunnyvale, USA) SpectraMax 190 microplate reader. Cell viability was calculated as the ratio of the absorbance from TCP-treated cells to that from cells incubated with culture medium only. All experiments were performed in triplicate and independently repeated three times.

### 2.6. Animal Handling

All animal experiments were conducted in accordance with protocols approved by the Institutional Animal Care and Use Committee (IACUC) of Jiangnan University Medical Center [Approval Number: (2024)Y-68]. Female C57BL/6N (6–8 weeks) were purchased from Changzhou Cavens Laboratory Animals Co., Ltd. (Changzhou, China).

### 2.7. Biosafety Analysis

Healthy C57BL/6N female mice were randomly divided into two groups (*n* = 3 per group), including a control group (PBS-treated) and an experimental group (TCP-treated). To collect the blood for blood routine and biochemistry analysis, the mice were sacrificed 7 days after intravenous injection. In addition, the main organs of mice were collected for hematoxylin & eosin (H&E) staining analysis by 3DHistech (Budapest, Hungary) Pannoramic MIDI system.

### 2.8. Hemolysis Assay

Whole blood was collected from healthy C57BL/6N mice via orbital venous plexus puncture and centrifuged at 2000 rpm for 10 min to isolate red blood cells (RBCs). The RBCs were washed three times with PBS and resuspended to obtain a 5% (*v*/*v*) RBC stock suspension in 1× PBS. 200 µL of the 5% RBC suspension was mixed with an equal volume of TCP solutions (5, 10, 20, 40, 60, 80, and 100 µg/mL), PBS (negative control), or distilled water (positive control). After incubation at 37 °C for 2 h, the mixtures were centrifuged at 12,000 rpm for 2 min, and 100 µL of the supernatant was transferred to a 96-well plate. The absorbance at 540 nm was measured using a microplate reader, and the hemolysis rate was calculated as: Hemolysis (%) = (OD_test − OD_negative)/(OD_positive − OD_negative) × 100%.

### 2.9. NIR-II Imaging of Vasculature in Mice

Healthy C57BL/6N mice were removed the hair on the abdomen and head after anesthesia. Upon injection of ICG or TCP (100 μL, 1 mmol/L in normal saline) through the tail vein, the mice were immediately placed in a supine position under isoflurane anesthesia. The NIR-II signal was collected under a series of Thorlabs (Newton, NJ, USA) long-pass (LP) filters upon excitation at 808 nm (30 mW/cm^2^) with varying exposure times.

### 2.10. SBR Calculation

For whole-body vascular imaging, SBR was quantified using a multi-point sampling method. Briefly, the distinct anatomical vessels (e.g., femoral artery/vein, carotid artery, abdominal aorta, and middle cerebral artery) were selected as representative signal regions, while the adjacent subcutaneous tissue or muscle, free from major organs, were chosen as background regions. The mean fluorescence intensity (I) was measured within each region of interest (ROI). The average intensity from 3 representative vascular ROIs was defined as I_signal, and the average from 3 adjacent background ROIs as I_background. The SBR was calculated as: SBR = I_signal/I_background. The same principle of comparing vessel signal to immediately adjacent tissue background was applied for SBR calculations at selected individual vessels

### 2.11. Deep Learning Method

Each convolutional encoding module consists of a 2-D convolutional layer (Conv2D), a batch normalization layer (BatchNorm), and a subsequent ReLU activation function. The output channels for the convolutional layers are 32, 64, and 128, respectively, with a kernel size of 3 and a stride of 2. The Swin Transformer Module (STM) receives encoder features as input, partitioning them along channel dimension into 5 × 5 non-overlapping patches. These patches are subsequently flattened into 1-D vectors and are position-embedded using a linear layer. The positionally encoded features are fed into three Swin Transformer blocks for further feature interaction, yielding re-mapped 1-D features. These re-mapped features are concatenated and reconstructed to form an encoded feature with global characteristics, which serves as the initial input for the convolutional decoding modules. The decoding modules up-sample the input features, concatenate them with the features from the skip connections, and then perform a sequence of convolution, batch normalization, and activation operations. The convolutional parameters mirror those of the encoding modules but use a stride of 1. Finally, the convolutional decoding modules reconstruct the predicted image, with an expected quality comparable to that of LP1300.

### 2.12. Statistical Analysis

Data are presented as the mean ± standard deviation (SD). Statistical analysis was performed using IBM SPSS Statistics 27 or OriginPro 2024b. Comparisons between two groups were conducted using Student’s *t*-test. Significance levels were defined as * *p* < 0.05, ** *p* < 0.01, and *** *p* < 0.001.

## 3. Results and Discussion

### 3.1. Comparison of Optical Performance Between TCP and ICG

**Figure 1:**
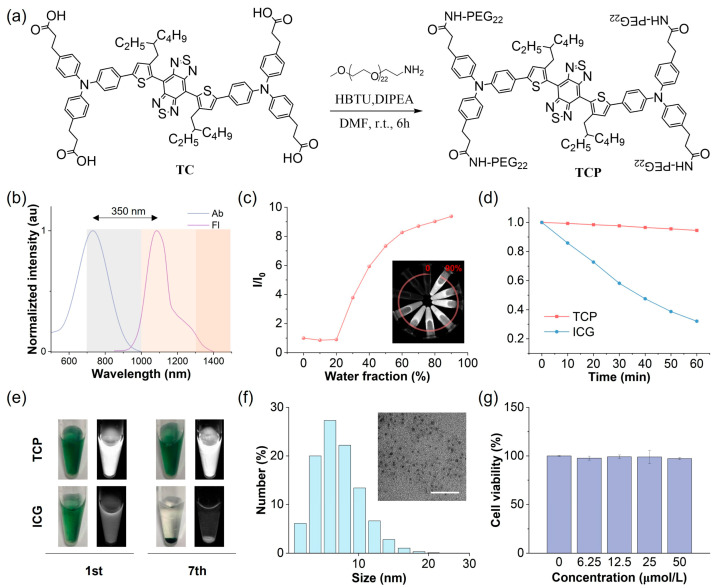
Chemical structure and physical and biochemical properties of TCP. (**a**) Synthetic route and chemical structure of TCP. (**b**) Normalized absorption (Ab) and fluorescence (Fl) spectra of TCP in water. (**c**) Changes in relative emission intensity (I/I_0_) versus the water fraction of TCP in N,N-dimethylformamide. (**d**) Changes in emission intensity for TCP and ICG in water under continuous 808 nm laser irradiation (60 min, 1 W/cm^2^). (**e**) Bright-field (left) and NIR-II fluorescent (right) images of TCP and ICG in water (10 μmol/L) on days 1 (1st) and 7 (7th). (**f**) Dynamic light scattering (DLS) analysis of hydrodynamic diameter and transmission electron microscopy (TEM) image (scale bar: 100 nm) of TCP in water. (**g**) Viability of 293 cells after 24 h incubation with TCP at indicated concentrations.

ICG is the most widely employed NIR fluorescent imaging probe in clinical practice, with a maximum absorption peak at 796 nm and a maximum emission peak at 854 nm in aqueous solutions, resulting in a relatively short Stokes shift of approximately 58 nm (Figure S1). The primary emission spectrum of ICG is situated within the NIR-I region, with a minor emission tail extending beyond 1000 nm into the NIR-II window. It is well established that the NIR-II region, particularly the NIR-IIa region beyond 1300 nm, improves the SBR of fluorescence imaging. Consequently, by harnessing the high fluorescence brightness characteristic of AIE probes, we aimed to develop NIR-II AIE fluorescent probes with extended emission wavelengths and increased brightness. Benzo[1,2-c:4,5-c′]bis [1,2,5]thiadiazole (BBTD) was utilized as the electron acceptor, while carboxyl-substituted triphenylamine served as the electron donor and AIE-active molecular rotor (Figure 1a and Figure S2). Theoretical calculations can be used to provide detailed mechanistic and dynamic insights into molecules transitioning between their ground (S_0_) and excited (S_1_) states and to investigate their photophysical properties. The alkyl substitution on the thiophene unit induced a significant cross-sectional angle (~50°) between the thiophene and BBTD core, effectively inhibiting intermolecular π-π stacking and mitigating nonradiative energy loss (Figure S3). The electron density of the highest occupied molecular orbital (HOMO) was concentrated along the conjugated backbone, while the lowest unoccupied molecular orbital (LUMO) extended from the acceptor core to the alkyl-substituted thiophene unit (Figure S4). This narrow energy gap implies a redshift in the emission wavelength of the probe. The main nonradiative decay pathway of an excited molecule is closely associated with its reorganization energy, defined as the relaxation energy from the S_1_ HOMO to the S_0_ HOMO [29]. TCP’s total reorganization energy was determined to be 1574.49 cm^−1^, comprising a 39.5% contribution from the bond length, 37.0% from the bond angle, and 23.5% from the dihedral angle (Figure S5). This low total reorganization energy suggests that the nonradiative decay pathway can be effectively suppressed by minimizing intramolecular motions.

To address the inherent hydrophobicity of the NIR-II AIE probe, we conjugated hydrophilic polyethylene glycol (PEG) chains to the carboxyl groups of TC to obtain the amphiphilic construct TCP, significantly enhancing its systemic bioavailability (Figures S6–S9). In aqueous environments, TCP exhibits a maximum absorption at 731 nm and a peak emission at 1081 nm within the NIR-II window (Figure 1b). Its notably large Stokes shift (350 nm) can effectively reduce background autofluorescence and minimize spectral bleed-through from the excitation source. Importantly, TCP maintains a pronounced emission tail beyond 1300 nm, within the NIR-IIa region, allowing for unprecedented penetration depth and spatial resolution in transcranial fluorescence imaging. Analysis of the optical profiles of TCP in N,N-dimethylformamide with varying water fractions showed a moderately reduced emission intensity, with the emission peaks redshifted below 20% water content due to the twisted intramolecular charge transfer effect, while the intensity significantly increased from 30% to 90% as a result of the dominant AIE effect (Figure 1c and Figure S10). Moreover, the emission wavelengths and intensity of TCP remained stable under different pH values or solvent concentrations (Figures S11 and S12). To investigate aggregation behaviors, molecular dynamics simulations were performed using Gromacs software 2022.4 (Figure S13). At the beginning of the simulation, the TCP molecules were well dispersed, with primarily extended linear conformations, reflecting their unrestricted motions. As the simulation proceeded, the molecules rapidly reorganized and gradually assembled into aggregates. By 50 ns, distinct aggregate structures had formed within the system, and thereafter, their overall morphology remained largely stable, sustaining a dynamic yet equilibrated state characterized by local conformational fluctuations. This result indicates restricted intramolecular motion upon aggregation, consistent with the pronounced AIE behavior of TCP. To sum up, TCP integrates bathochromic NIR-II emissions with robust AIE activity, demonstrating its suitability for deep-tissue and high-contrast imaging.

### 3.2. In Vitro and In Vivo Performance of TCP

The stability of fluorescent probes is critical for their application in biological studies and clinical translation. Initially, we evaluated the photostability of TCP in comparison to the clinical gold-standard dye ICG under continuous 808 nm irradiation under aqueous conditions (Figure 1d). While ICG exhibited a reduction of over 70% compared to its initial emission, TCP maintained its NIR-II fluorescence intensity. We also assessed the fluorescence stability of TCP and ICG in an aqueous medium after long-term storage. TCP demonstrated stable emission and dispersion, attributed to its AIE characteristics and amphiphilic nature (Figure 1e). Conversely, ICG showed precipitation and fluorescence attenuation due to its own hydrophobicity. These findings illustrate that TCP possesses superior optical properties and stability for biological applications compared to the clinically utilized agent ICG. Owing to these favorable properties, we next examined the morphological state and biosafety of TCP. Dynamic light scattering (DLS) analysis revealed that its hydrodynamic diameters mainly ranged from 5 to 8 nm, while transmission electron microscopy (TEM) confirmed that TCP forms in spherical shapes (Figure 1f). Moreover, similar to its status in water, TCP particle size was consistently around 7 nm in PBS and FBS (Figure S14). These results suggest that TCP can spontaneously self-assemble in water to form ultra-small and uniform organic dots, facilitating internal circulation and improving bioavailability.

Biosafety is another crucial indicator of probe potential in biological applications. Thus, we investigated the in vitro and in vivo toxicity of TCP prior to in vivo fluorescent angiography. A standard MTT assay was performed to assess the cytotoxicity of TCP on normal 293 cells in vitro. Cell viability exceeded 95% following incubation with TCP at a concentration of 50 μmol/L, suggesting that TCP does not induce significant in vitro toxicity (Figure 1g). To assess the potential hematological impact of TCP upon entering the circulation, we evaluated its hemocompatibility by performing an in vitro hemolysis assay with RBCs. TCP did not cause significant hemolysis at concentrations up to 100 μg/mL (Figure S15), with a hemolysis rate of 4.37% at the highest concentration tested; this remains below the 5% safety threshold defined by the ASTM E2524-08 standard, confirming that TCP does not damage erythrocytes and exhibits favorable blood compatibility. To further validate the in vivo biosafety of TCP, biochemical and hematological analyses were conducted on samples collected from healthy C57BL/6N female mice administered either 200 μL of TCP (1 mmol/L) or normal saline as a control for 7 days. In comparison with the control group, the TCP-treated mice exhibited no significant differences in any blood parameters (Figure S16); additionally, hematoxylin and eosin (H&E) staining revealed no abnormalities in the major organ tissues of TCP-treated mice (Figure S17). Overall, these results indicate that TCP possesses low cytotoxicity and favorable biocompatibility, making it suitable for imaging applications in in vivo studies.

**Figure 2:**
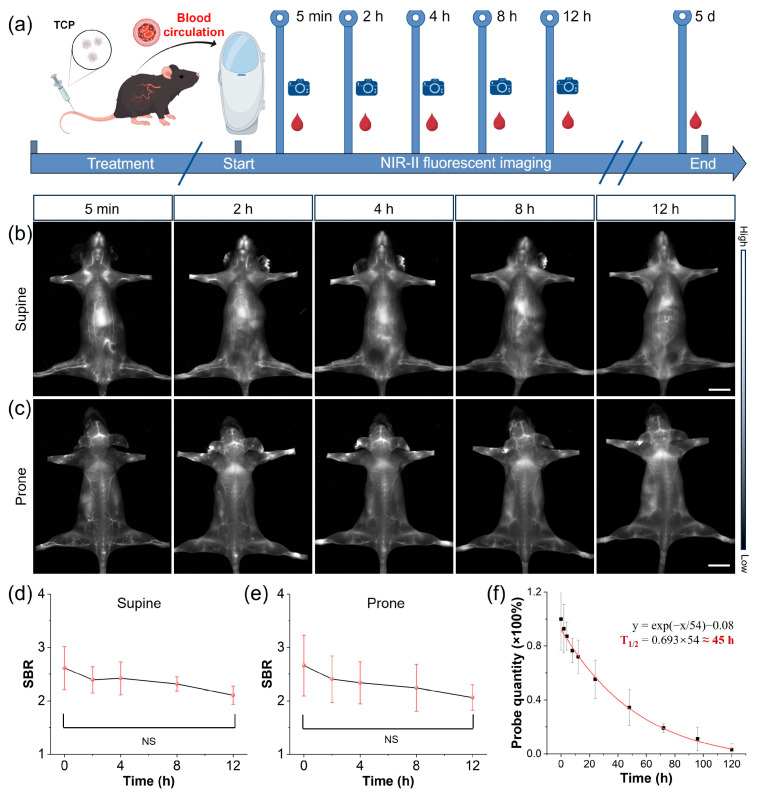
NIR-II imaging of long-circulating TCP. (**a**) Schematic representing TCP’s long vascular circulation, monitored using in vivo NIR-II imaging and ex vivo blood analysis at indicated post-injection time points. (**b**) Representative in vivo NIR-II fluorescence images of TCP-treated mouse in supine position at different time points under 808 nm laser irradiation (filter: LP1300 nm, exposure time: 500 ms), scale bar = 1 cm. (**c**) Representative in vivo NIR-II fluorescence images of TCP-treated mouse in prone position at different time points under 808 nm laser irradiation (filter: LP1300 nm, exposure time: 500 ms), scale bar = 1 cm. (**d**) Time-dependent SBR quantification of average fluorescent angiography of supine TCP-treated mice at different time points (mean ± s.d., *n* = 3). (**e**) Time-dependent SBR quantification of average fluorescent angiography of prone TCP-treated mice at different time points (mean ± s.d., *n* = 3); (**f**) Ex vivo quantitative analysis of TCP in mice blood at different time points (mean ± s.d., *n* = 3).

Prolonged blood circulation is a pivotal determinant of diagnostic accuracy and safety, representing a key translational breakthrough for moving contrast agents from bench to bedside. Thus, we evaluated the vascular circulatory capacity of TCP by intravenously injecting healthy C57BL/6N female mice (*n* = 3) with TCP (200 μL, 1 mmol/L), subsequently monitoring them using an in vivo NIR-II imaging system (Figure 2a). Blood samples were collected at various time points using capillary tubes, with TCP demonstrating an extended blood circulation time attributable to the stealth properties conferred by its chains (Figure 2b,c and Figure S18) [30]. Blood vessel SBR values in fluorescent angiography serve as another crucial metric for assessing imaging quality. Upon quantifying blood vessel signals at various time points, the SBR values for TCP-treated mice remained above 2.0 for up to 12 h (Figure 2b,e). In addition, when quantifying the blood circulation time of TCP, we estimated its circulation half-life to be approximately 45 h. By the fifth day post injection, fluorescence in the blood had decreased to less than 5% of the injected dose (Figure 2f). This extended retention time could facilitate long-term, precise monitoring of blood vessels and enhance the accumulation of the fluorophore in targeted tissues. To further elucidate the biodistribution of TCP in mice, ex vivo NIR-II fluorescence imaging was conducted on organs harvested 24 h after the intravenous injection of TCP. Predominant uptake of TCP was shown in the liver, indicating that the hepatobiliary pathway is its primary metabolic pathway (Figure S19). The high brightness and prolonged circulation of TCP enable high-resolution whole-body vessel imaging, prompting further investigation into vascular-related disorders.

### 3.3. Comparison of TCP and ICG via In Vivo Fluorescent Angiography

**Figure 3:**
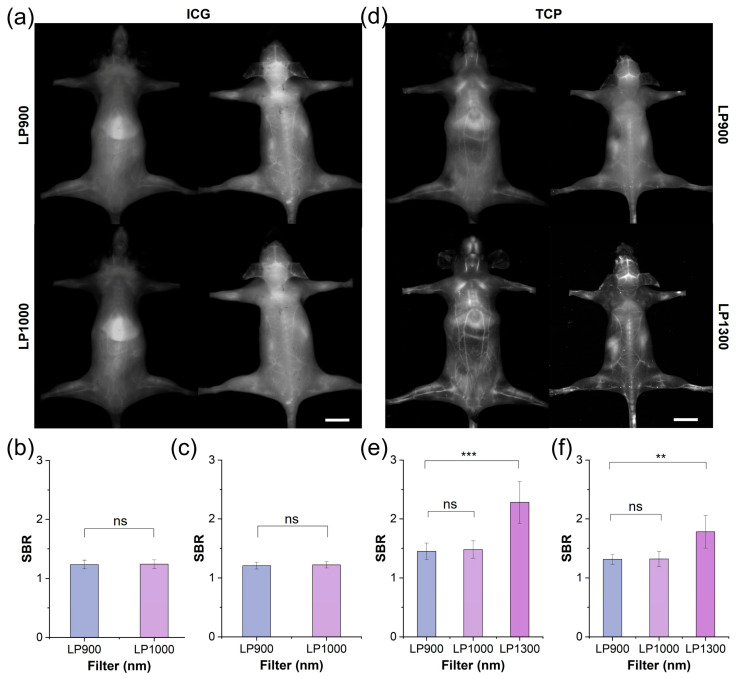
NIR-II imaging and SBR analysis of TCP and ICG under different filters. (**a**) Fluorescent angiography of representative mouse in supine and prone positions after intravenous injection of ICG (200 μL, 1 mmol/L in 1× PBS) under LP900 (exposure time: 5 ms) and LP1000 filters (exposure time: 10 ms), scale bar: 1 cm. (**b**) SBR of blood vessels in supine mouse shown in (**a**). (**c**) SBR of blood vessels in prone mouse shown in (**a**). (**d**) Fluorescent angiography of representative mouse in supine and prone positions after intravenous injection of TCP (200 μL, 1 mmol/L in 1× PBS) under LP900 (exposure time: 5 ms) and LP1300 filters (exposure time: 500 ms), scale bar: 1 cm. (**e**) SBR of blood vessels in supine mouse shown in (**d**); (**f**) SBR of blood vessels in prone mouse shown in (**d**). ns > 0.05, *** *p* < 0.001, ** *p*< 0.01.

Motivated by the advantageous optical properties and biocompatibility of TCP, we compared its performance with that of ICG under in vivo fluorescence angiography. After intravenous administration of ICG or TCP (100 μL, 1 mmol/L in saline), the whole-body vasculature of C57BL/6NJ mice was illuminated under 808 nm laser irradiation. In the ICG-treated group (Figure 3a), the whole-body blood vessel signals were relatively weak in both the supine and prone positions under the long-pass 900 nm (LP900) filter of the NIR-I window, with a pronounced signal observed in the liver. Although ICG exhibited an emission tail for traditional NIR-II imaging under the long-pass 1000 nm (LP1000) filter, the vascular signals did not show significant improvement to allow clear observation. Quantitative analysis further confirmed that the SBR values for both filters remained below 1.5, with no statistically significant difference observed between them (Figure 3b,c). These results indicate that ICG’s emission tail beyond 1000 nm does not enhance the quality of vascular imaging. In contrast, whole-body fluorescent angiography was clearer in the TCP-treated group than in the ICG-treated group under both the LP900 and LP1000 filters (Figure 3d and Figure S20). Notably, under the long-pass 1300 nm (LP1300) filter, the whole-body blood vessel signals were significantly improved, while background signals from skin tissue were effectively reduced; additionally, greater detail was shown for small blood vessels, while the signal from liver tissue was reduced. Motivated by the high-quality imaging results obtained beyond 1300 nm, we quantified the SBRs of blood vessel signals under the three filters: under the LP1300 filter, SBR values reached 2.5, significantly higher than under the other two filters (Figure 3e,f). TCP can be used for high-contrast vascular imaging due to its PEGylated design, which ensures prolonged circulation and reduced liver uptake, in addition to its inherently low-background NIR-IIa imaging window, which minimizes tissue autofluorescence and scattering. Thus, TCP outperformed the clinical standard ICG by providing substantially brighter and sharper whole-body vascular images in the NIR-IIa window while simultaneously suppressing liver and skin background signals, resulting in SBR values that were more than 2-fold higher than ICG and confirming its superiority for NIR-IIa fluorescence angiography.

**Figure 4:**
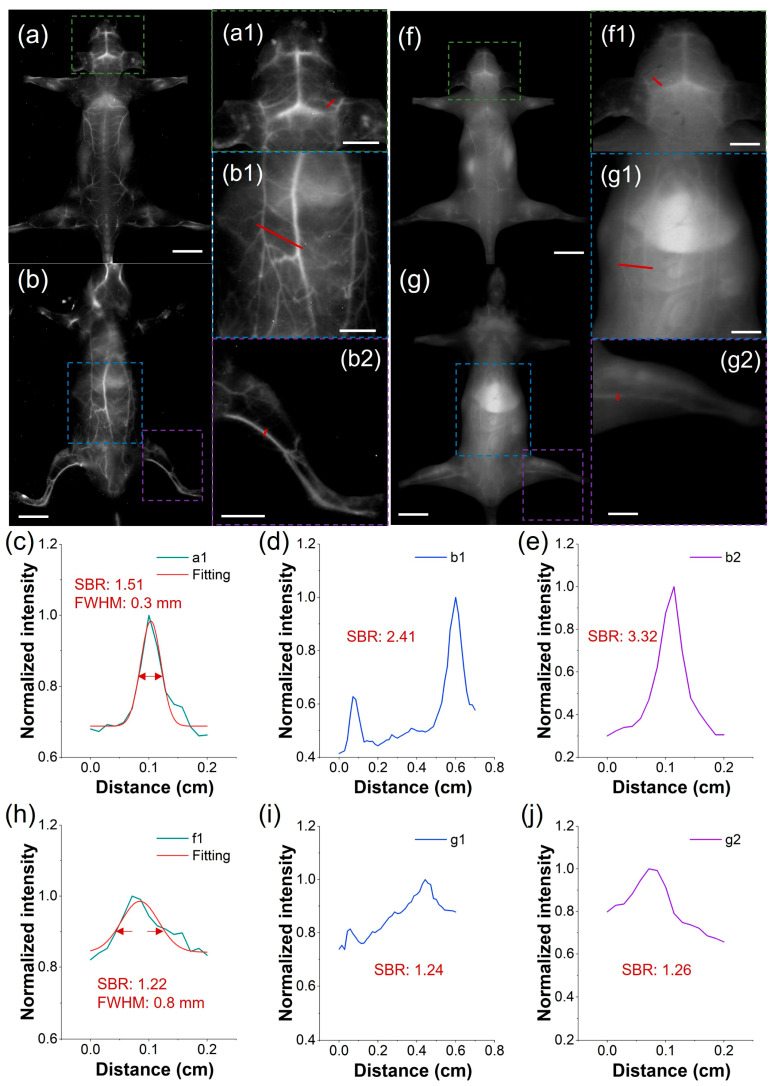
Comparison of NIR-II imaging between TCP and ICG. (**a**,**b**) Fluorescence angiography of a representative mouse after intravenous injection of TCP (100 μL, 1 mmol/L) in prone and supine positions (filter: LP1300 nm, exposure time: 500 ms, scale bar: 1 cm). (**c**–**e**) Cross-sectional fluorescence intensity profiles of blood vessels at the mouse head, abdomen, and leg highlighted by the red lines in (**a1**,**b1**,**b2**). (**f**,**g**) Fluorescence angiography of a representative mouse after intravenous injection of ICG (100 μL, 1 mmol/L) in prone and supine positions (filter: LP1000 nm, exposure time: 500 ms, scale bar: 1 cm). (**h**–**j**) Cross-sectional fluorescence intensity profiles of blood vessels at the mouse head, abdomen, and leg highlighted by the red lines in (**f1**,**g1**,**g2**). (Green, blue, and purple boxes represent the partially enlarged details of the mouse head, abdomen, and leg, respectively; scale bar: 0.5 cm).

To address the clinical demand for detailed vascular imaging, we compared the high-resolution angiographic capabilities of both TCP and ICG after intravenous injection, focusing on the quantification of SBR and full-width-at-half-maximum (FWHM) in cerebral and peripheral vessels. Under the LP1300 filter, TCP-treated mice exhibited well-defined vasculature in the head, back, abdomen, and limbs (Figure 4a,b). In contrast, under the LP1000 filter, the vessels of ICG-treated mice were nearly undetectable due to pronounced skin autofluorescence (Figure 4f,g). Furthermore, we measured blood vessel SBRs in the representative mice by analyzing partially enlarged images from the in vivo fluorescent angiography. In mice injected with TCP, SBRs were as high as 3.32, whereas in mice injected with ICG, they remained below 1.26, demonstrating a 2.6-fold greater SBR in TCP under NIR-IIa imaging than that of ICG under traditional NIR-II imaging (Figure 4c–e,h–j). This demonstrates the benefit of the low background signal under the LP1300 filter, in contrast to conventional NIR-II imaging with the LP1000 filter. Furthermore, the microvasculature in the representative mouse head after TCP treatment demonstrated an accurate FWHM of 0.3 mm (Figure 4c) and distinct tiny vessels compared to the ICG-treated group, with an FWHM only accurate to 0.8 mm (Figure 4h). Overall, these findings demonstrate that TCP, with its unique emission range beyond 1300 nm, significantly enhances in vivo fluorescence angiography compared to the clinical standard ICG probe. Nevertheless, extended exposure times were necessary to acquire high-quality images; developing strategies to reduce acquisition time while maintaining image quality is therefore a critical priority in order to facilitate real-time imaging.

### 3.4. Applying Deep Learning to In Vivo NIR-IIa-like Imaging

**Figure 5:**
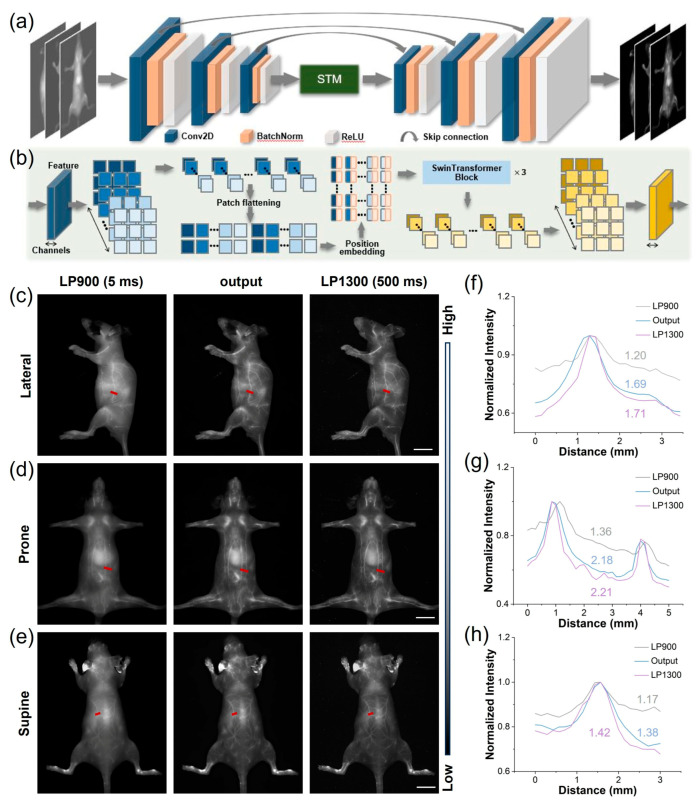
Application of deep learning to images of TCP treatment using both NIR-I and NIR-IIa. (**a**) End-to-end deep learning workflow for enhancement of fluorescent images. (**b**) Architecture of the image-transformation-guided distillation model. (**c**–**e**) Representative mouse angiograms acquired through LP900 (5 ms exposure) and LP1300 (500 ms exposure) filters simultaneously, alongside STM-generated images from the LP900 input in prone, supine, and lateral positions (scale bar, 1 cm). (**f**–**h**) Corresponding cross-sectional fluorescence intensity profiles along the red dashed lines in (**c**–**e**), demonstrating the recovered vessel sharpness and contrast.

In the realm of biomedical imaging, deep learning-based image post-processing is a pivotal enhancement technique. These approaches enhance NIR-II imaging by transforming suboptimal low-resolution images into high-resolution versions. Deep learning can also improve probe imaging speed under large filter wavelengths, such as LP1300, which shortened the imaging time by two orders of magnitude. Therefore, we utilized LP900–1300 filters to obtain intrinsically co-registered pairs of high- and low-quality images from NIR-I and NIR-II cameras capturing images of TCP-treated mice simultaneously. These pairs were used to construct a database for training a SwinUnet image enhancement neural network specifically aimed at improving LP900 imaging quality. The SwinUnet framework was designed to enhance fluorescence imaging (Figure 5a). Its backbone comprises three convolutional encoding modules, a Swin Transformer Module (STM), and three convolutional decoding modules. The images acquired using LP1300 and LP900 exhibited a high degree of structural similarity; therefore, inherently localized convolutional operations were employed to extract local structural features from the LP900 images. These features were propagated to the decoding pathway through skip connections. The STM captures spatial correlations within the convolutional feature maps, thereby generating global fine features (Figure 5b). The dense interactions between global features provided higher-dimensional information, enabling the decoding of high-quality structures and fine features.

We processed LP900 (NIR-I) images of mice in multiple positions using the trained STM network, resulting in high-fidelity reconstructions that were subsequently compared with their ground-truth LP1300 (NIR-IIa) counterparts. The original LP900 images displayed indistinct subdermal vasculature, whereas the STM-enhanced images revealed a sharply delineated whole-body vascular network closely approximating the reference LP1300 images (Figure 5c–e); notably, despite this approximation, the images are not identical, indicating that the neural network did not simply memorize the results from the training set but also effectively generalized to previously unseen data. Subsequently, we performed SBR analysis on the same images (Figure 5f–h). The results demonstrated that the generated images exhibited significantly improved SBR values compared to the original NIR-I images. Furthermore, the SBRs of the generated images were generally close to those of the LP1300 images. These findings suggest that the STM neural network enhances the clarity and spatial resolution of NIR-I images through transformation, making them akin to long-wavelength NIR-IIa images.

### 3.5. Transforming NIR-I ICG Images to NIR-IIa-like Images

**Figure 6:**
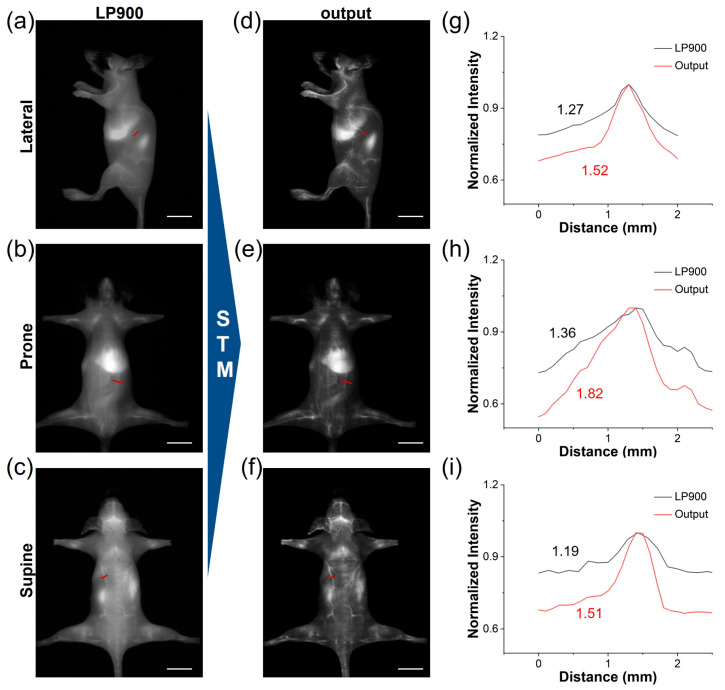
Deep learning of ICG from NIR-I to NIR-IIa. (**a**–**c**) NIR-I fluorescence images of a representative ICG-treated mouse acquired simultaneously from LP900 filter (exposure time: 5 ms, scale bar: 1 cm). (**d**–**f**) STM-transformed NIR-IIa-like images, corresponding to (**a**–**c**) (scale bar: 1 cm). (**g**–**i**) Cross-sectional fluorescence intensity profiles of blood vessels according to the red lines in (**a**–**f**), illustrating the enhanced vessel contrast achieved by deep learning reconstruction.

Utilizing the deep learning model trained on our STM dataset, we translated the conventional NIR-I images of ICG-injected mice into high-fidelity NIR-IIa-like angiograms. Under the LP900 filter, the initial NIR-I frames were predominantly affected by diffuse skin autofluorescence and intense hepatic signals, which obscured the vascular signals (Figure 6a–c). Following network inference, these frames were transformed into sharp, low-background images. The quality of the generated NIR-IIa-like images was significantly improved in comparison with the original images (Figure 6d–f). Notably, the whole-body vascular signals of ICG-treated mice in different positions became distinct due to the effective reduction in background signals. This approach offers the advantage of maintaining the rapid acquisition speed of NIR-I imaging while providing the contrast typically associated with authentic NIR-IIa imaging. We further conducted a comparative analysis of the SBR in blood vessels between the original NIR-I images and the deep learning-generated NIR-IIa-like images (Figure 6g–i). The SBR values for the generated images exceeded 1.5, demonstrating potential benefits for reliable intraoperative guidance. Therefore, the STM network can be used to convert low-contrast NIR-I angiograms into NIR-IIa-quality images, delivering sharp vessel delineation and an SBR boost of more than 1.5-fold without sacrificing the speed of NIR-I acquisition.

## 4. Conclusions

In conclusion, we have successfully developed an amphiphilic NIR-II AIE probe (TCP) and demonstrated its superior performance in enhancing the quality of fluorescent imaging relative to the clinical standard ICG. The distinctive optical properties of TCP, including its emission tail extending beyond 1300 nm, large Stokes shift, and exceptional stability, facilitate high-resolution in vivo fluorescent angiography with a 2.6-fold improvement in SBR in comparison with ICG. The self-assembled nanodots of TCP, with sizes of 5–8 nm, exhibit a circulation half-life of 45 h, thereby enabling long-term, high-contrast angiography. Furthermore, the incorporation of the SwinUnet deep learning framework effectively transforms low-definition NIR-I ICG images into high-resolution NIR-IIa-like images, addressing the limitations inherent in traditional NIR-I imaging. This study highlights the potential of integrating advanced NIR-II AIE probes with deep learning-based image processing to enhance clinical fluorescence imaging. This approach not only augments the clarity and spatial resolution of NIR-I images but also facilitates the rapid generation of NIR-IIa images for NIR-II AIE probes. This strategy has the potential to be applied to other fluorescent probes used in clinical practice, further expanding its utility in various biomedical applications.

Overall, our development of the TCP and deep learning-enhanced imaging approach represents a significant step forward in improving the quality of clinical fluorescence imaging. This work provides a new direction for the development of next-generation imaging probes and techniques that can facilitate more accurate diagnostics and image-guided surgeries in the clinic.

## Data Availability

The original contributions presented in this study are included in the article/Supplementary Material. Further inquiries can be directed to the corresponding authors.

## Supplementary Materials

The following supporting information can be downloaded at: https://www.mdpi.com/article/10.3390/bios16020090/s1, Figure S1: Normalized absorption (Ab) and fluorescence (Fl) spectra of ICG in water; Figure S2: The synthetic route of TC; Figure S3: Dihedral angles of TC in the S0 state calculated with time-dependent density functional theory (TD-DFT) at the level of cam-B3LYP/6-31G*; Figure S4: DFT-calculated HOMO and LUMO distributions of TC in the S0 and S1 states; Figure S5: The contribution to the total reorganization energy of TC from the bond stretch, band bend, and dihedral angle; Figures S6: 1H NMR spectrum of TC in DMSO-d6; Figure S7: 13C NMR spectrum of TC in DMSO-d6; Figure S8: 1H NMR spectrum of TCP in DMSO-d6; Figure S9: MALDI-TOF-MS spectrum of TCP; Figures S10: The absorption (a) and emission (b) spectra of TCP with the different water fractions (vol%) in DMF; Figure S11: The absorption (a) and emission (b) spectra of TCP with the different pH values; Figure S12: The absorption (a) and emission (b) spectra of TCP in water, PBS, and FBS; Figure S13: The progression of TCP in molecular dynamics simulation from 0 to 150 ns; Figure S14: The DLS analysis of TCP in water, PBS and FBS, respectively; Figure S15: Hemolysis assay of blood samples after incubated with TCP at 0-100 μg/mL; Figure S16: The blood biochemistry test and routine blood test results of samples collected from the mice inject with TCP; Figure S17: H&E staining slices of major organs for the representative mice treated with TCP probe or normal saline control, scale bar: 100 μm; Figure S18: The replicates of NIR-II fluorescence images of mice treated with TCP; Figure S19: The representative ex vivo NIR-II fluorescence imaging and quantitative analysis of different organs collected from mice treated with TCP; Figure S20: The fluorescent angiography of the representative mouse in supine position and prone position after intravenous injection of TCP.

## Abbreviations

The following abbreviations are used in this manuscript:

AIE	Aggregation-induced Emission
ICG	Indocyanine green
SBR	Signal-to-background Ratio
GAN	Generative Adversarial Network
ACQ	Aggregation-caused Quenching
HOMO	Highest Occupied Molecular Orbital
LUMO	Lowest Unoccupied Molecular Orbital
PEG	Polyethylene Glycol
STM	Swin Transformer Module
