# Supplementary material for: From the Clinic, to the Clinic: Improving the Fluorescent Imaging Quality of ICG via Amphiphilic NIR-IIa AIE Probe
Source: Biosensors (Basel). 2026 Feb 1;16(2):90. doi: 10.3390/bios16020090 (PMC12938000; doi:10.3390/bios16020090)
Supplement: Supplementary file 1 [file biosensors-16-00090-s001.zip › biosensors-4066510-supplementary.pdf]

Supporting Information

From the Clinic, to the Clinic: Improving the Fluorescent Imaging Quality of ICG via Amphiphilic NIR-IIa AIE Probe

Anjun Zhu [1,2], Zhibo Xiao [3], Aihui Sun [4], Feng Lu [1], Haozhou Tang [1,2,5], Xuekun Zhang [5], Ran Ren [1,2], Wei Yu [3], Andong Shao [5], Ninghan Feng [6,*], Shouyu Wang [3], Jianming Ni [2,*] and Yaxi Li [1,*]

[1] Department of Radiology, Jiangnan University Medical Center, Wuxi No. 2 People's Hospital, Wuxi 214002, China; zhuanjun_01@163.com (A.Z.); lufeng84@126.com (F.L.); tanghz1126@foxmail.com (H.T.); 6232841002@stu.jiangnan.edu.cn (R.R.)
[2] Department of Radiology, Wuxi 9th People's Hospital Affiliated to Soochow University, Wuxi 214062, China
[3] OptiX+ Laboratory, School of Electronics and Information Engineering, Wuxi University, Wuxi 214105, China; zhiboxiao2000@gmail.com (Z.X.); wei.yu@cwxu.edu.cn (W.Y.); shouyu29@cwxu.edu.cn (S.W.)
[4] Computational Optics Laboratory, School of Sciences, Jiangnan University, Wuxi 214122, China; aihuisun@jiangnan.edu.cn
[5] School of Life Sciences and Health Engineering, Jiangnan University, Wuxi 214122, China; 6233302050@stu.jiangnan.edu.cn (X.Z.); adshao@jiangnan.edu.cn (A.S.)
[6] Department of Urology, Wuxi No. 2 People's Hospital, Wuxi 214002, China
* Correspondence: n.feng@njmu.edu.cn (N.F.); nijianming@jiangnan.edu.cn (J.N.); liyx24001@jiangnan.edu.cn (Y.L.)

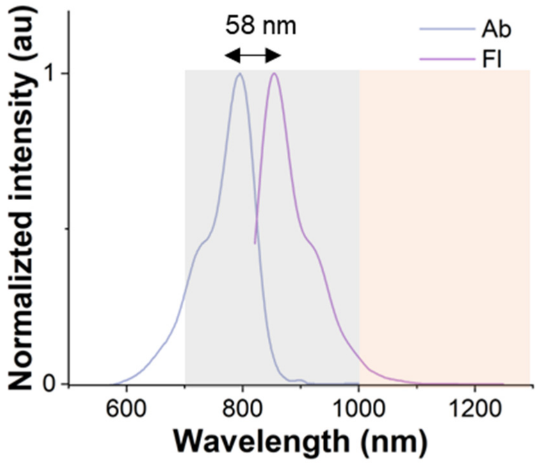

Figure. S1 Normalized absorption (Ab) and fluorescence (Fl) spectra of ICG in water.

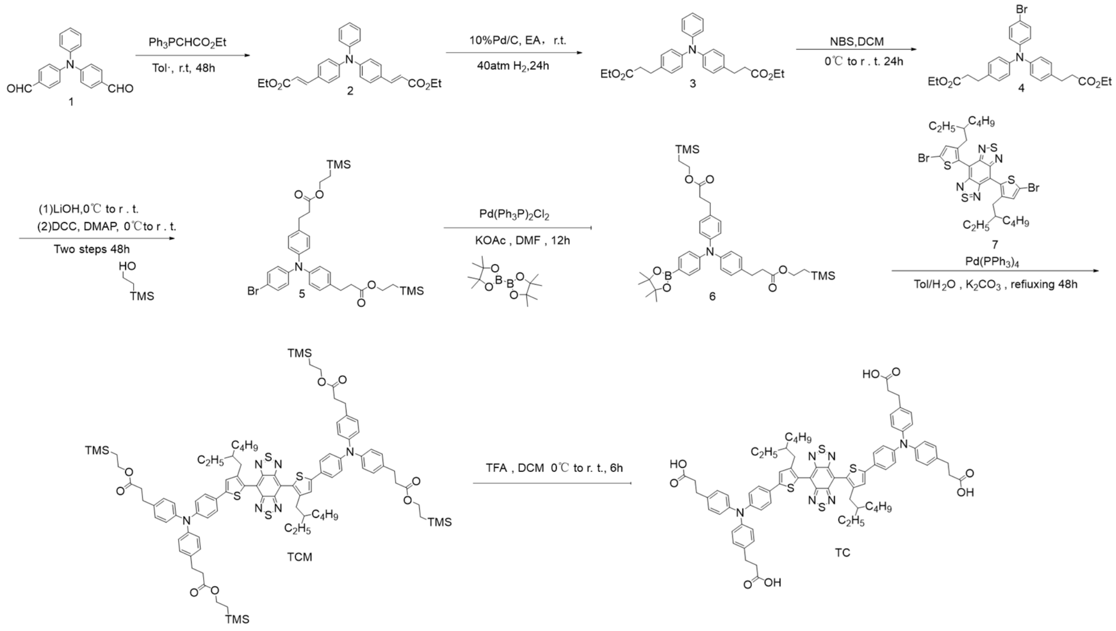

Figure. S2 The synthetic route of TC.

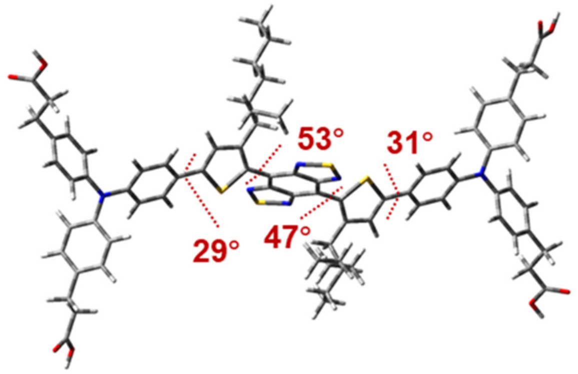

Figure. S3 Dihedral angles of TC in the S_0 state calculated with time-dependent density functional theory (TD-DFT) at the level of cam-B3LYP/6-31G*.

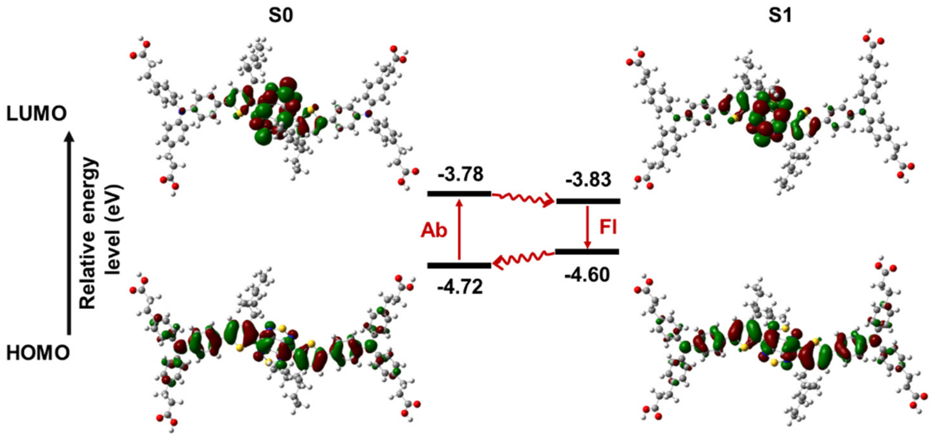

Figure. S4 DFT-calculated HOMO and LUMO distributions of TC in the S_0 and S_1 states.

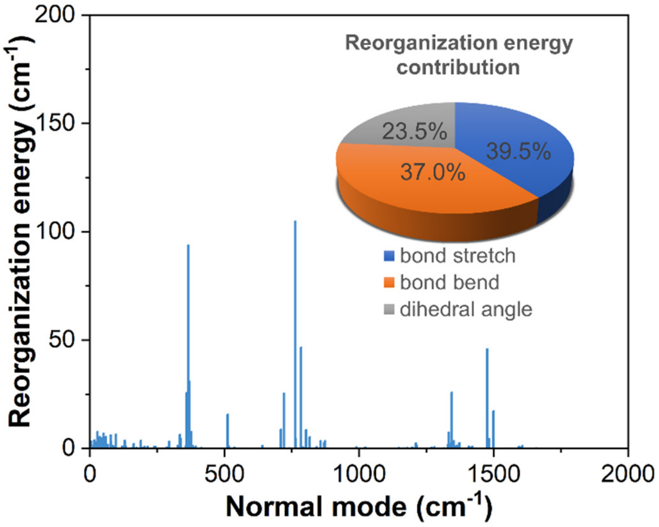

Figure. S5 The contribution to the total reorganization energy of TC from the bond stretch, band bend, and dihedral angle.

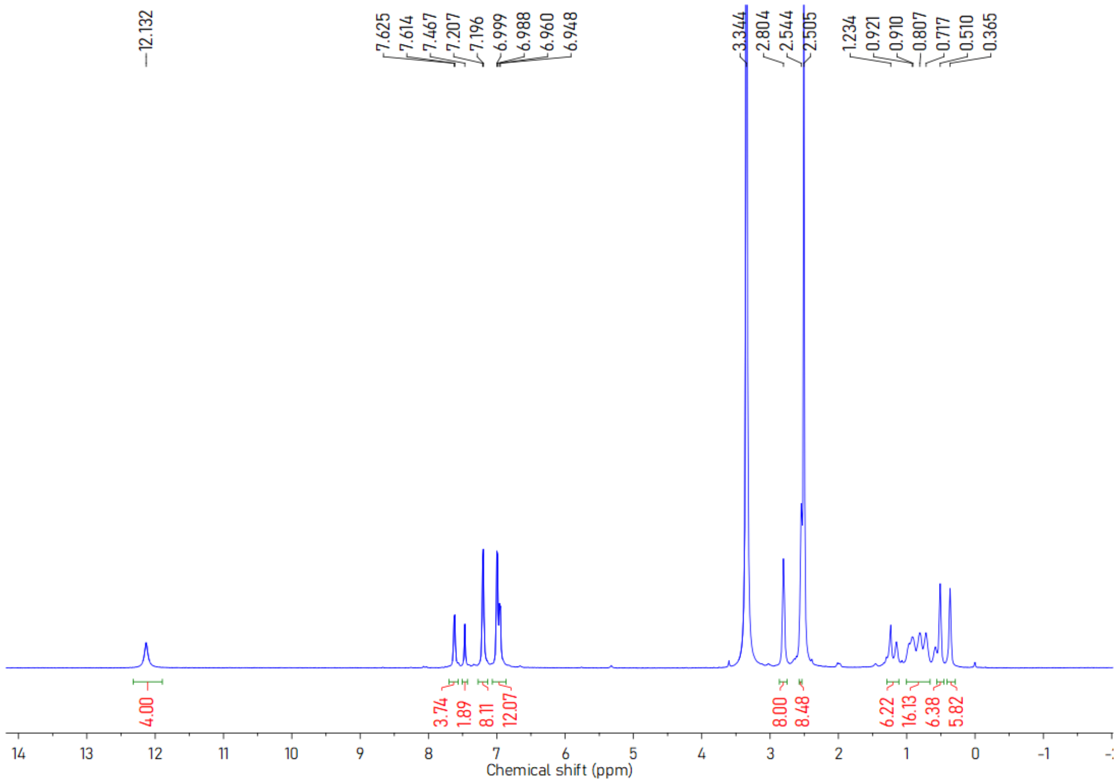

Figure. S6 ^{1}H NMR spectrum of TC in DMSO-d_6.

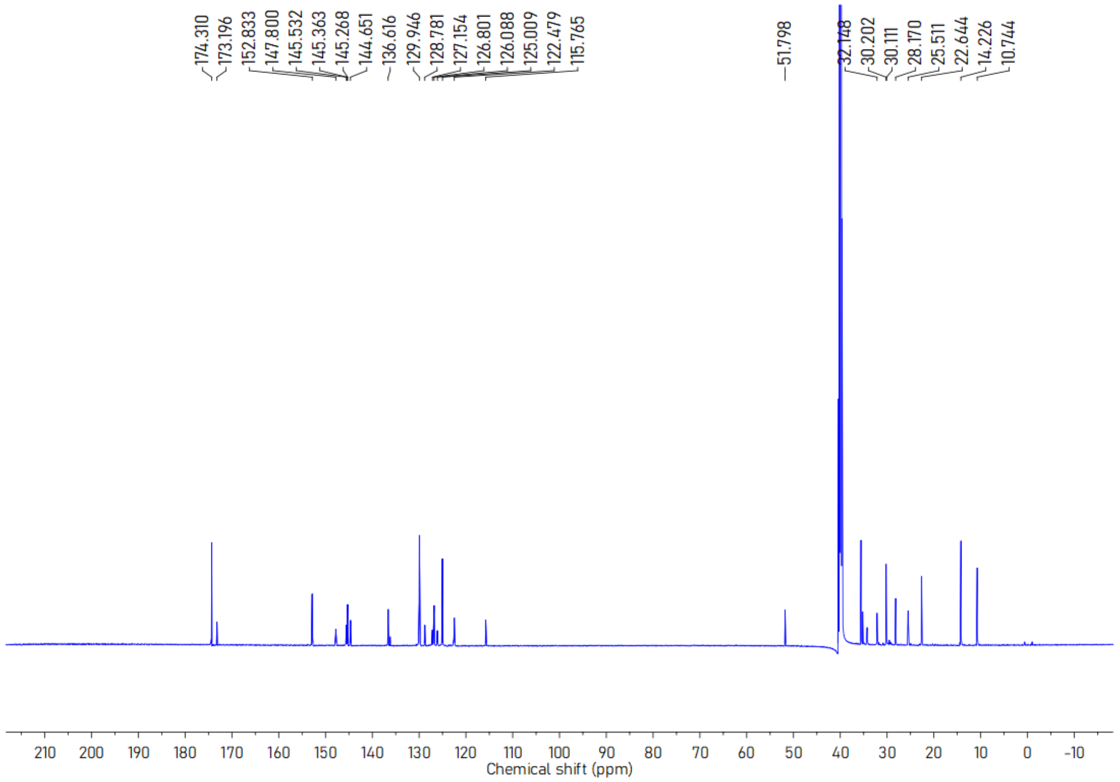

Figure. S7 ^{13}C NMR spectrum of TC in DMSO-d_6.

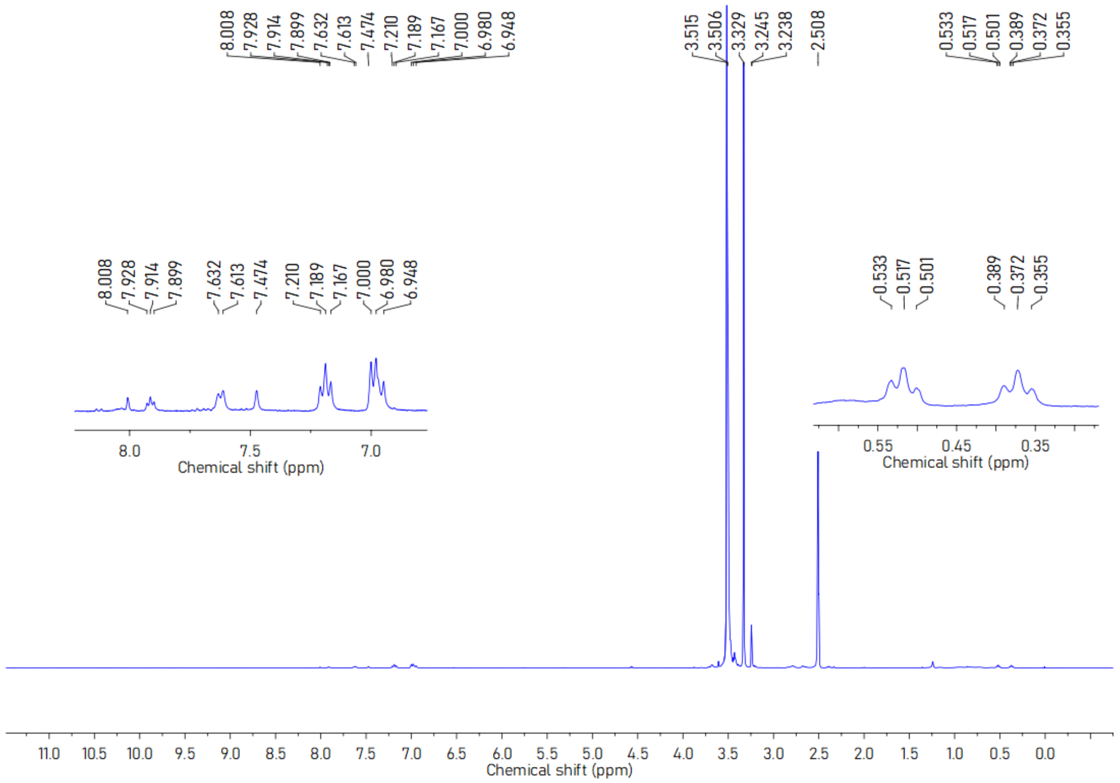

Figure. S8 ^{1}H NMR spectrum of TCP in DMSO-d_6.

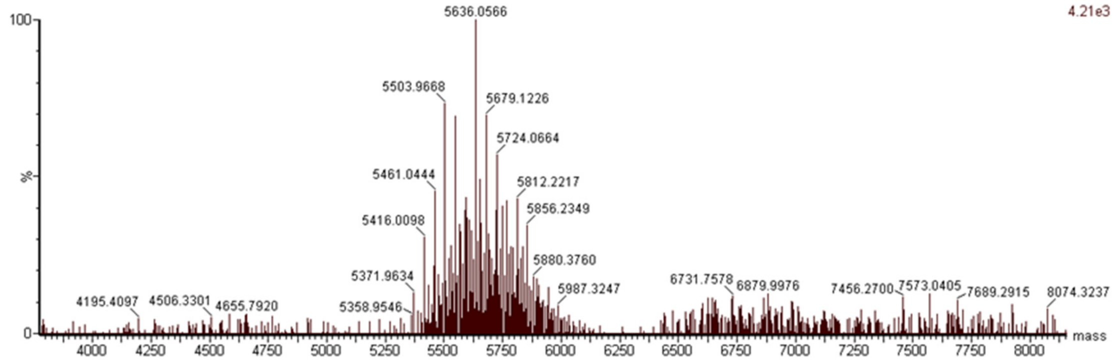

Figure. S9 MALDI-TOF-MS spectrum of TCP.

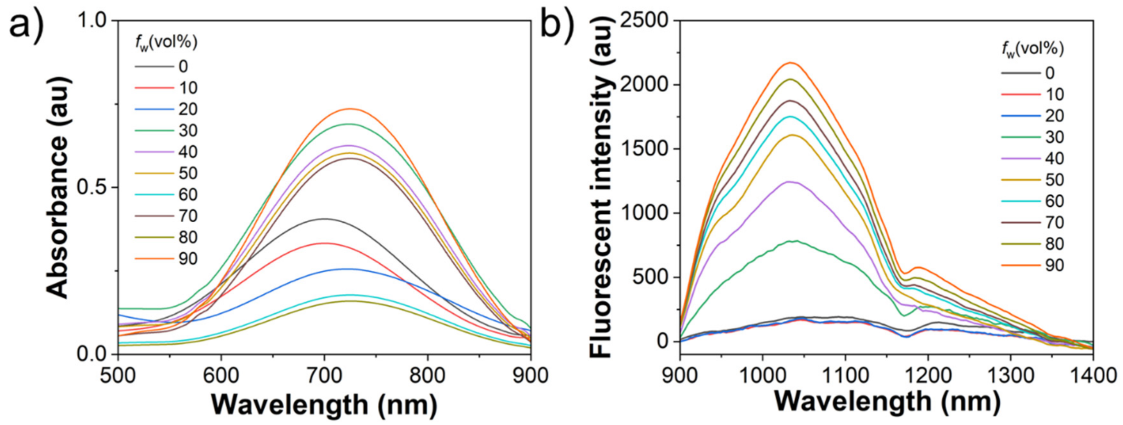

Figure. S10 The absorption (**a**) and emission (**b**) spectra of TCP with the different water fractions (vol%) in DMF.

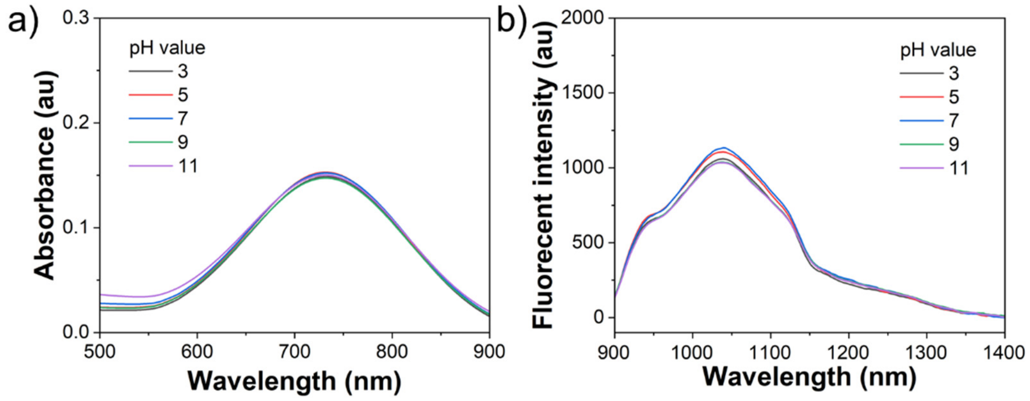

Figure. S11 The absorption (**a**) and emission (**b**) spectra of TCP with the different pH values.

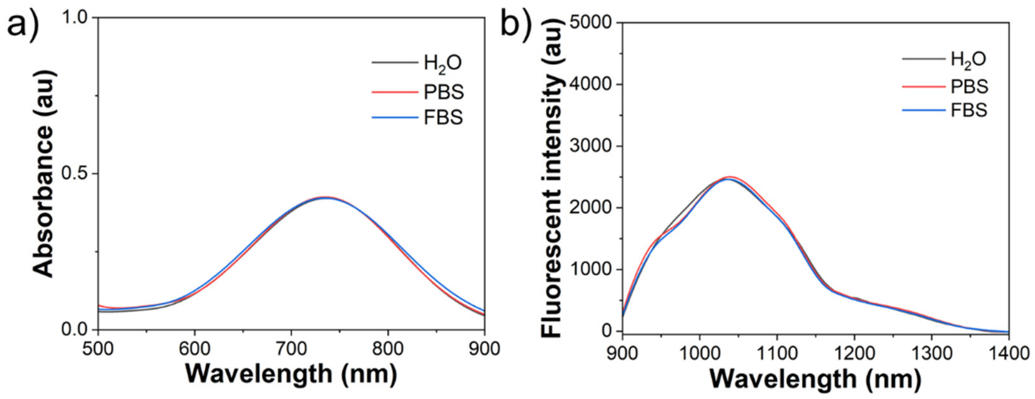

Figure. S12 The absorption (**a**) and emission (**b**) spectra of TCP in water, PBS, and FBS.

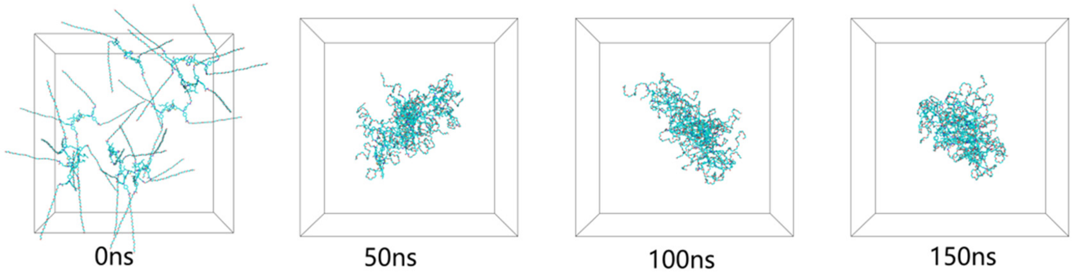

Figure. S13 The progression of TCP in molecular dynamics simulation from 0 to 150 ns.

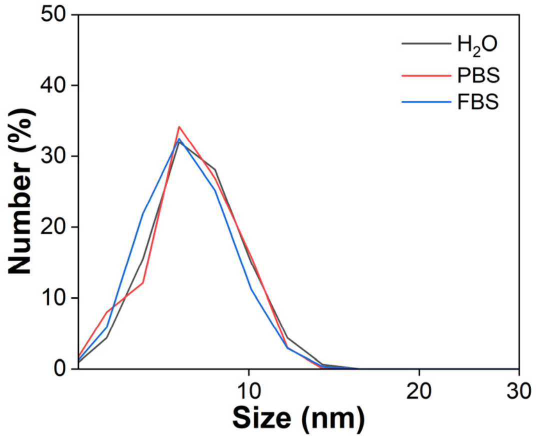

Figure. S14 The DLS analysis of TCP in water, PBS and FBS, respectively.

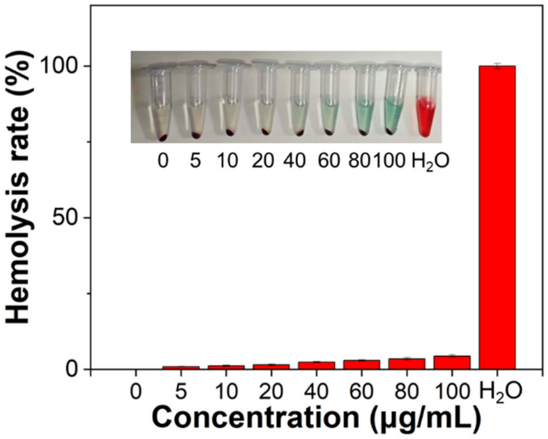

Figure. S15 Hemolysis assay of blood samples after incubated with TCP at 0-100 μg/mL.

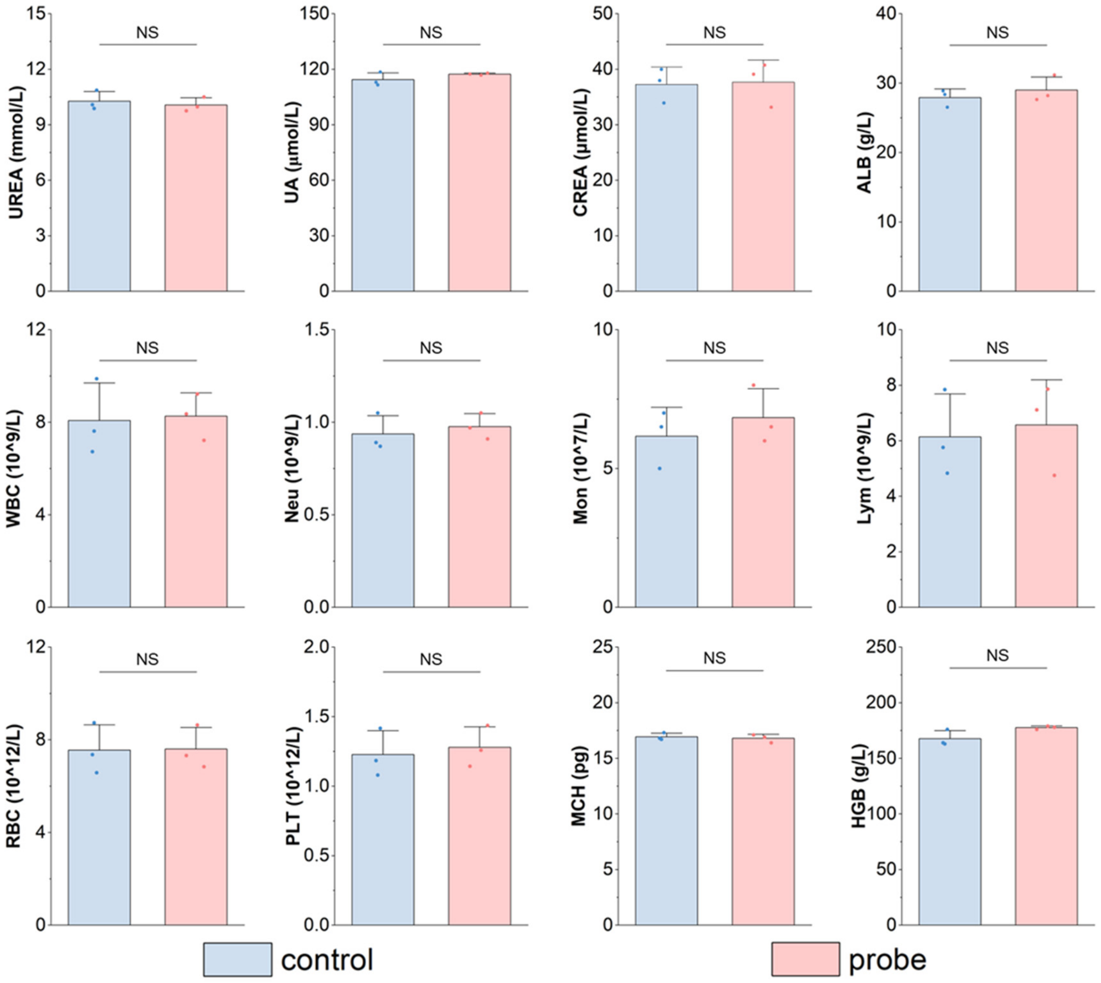

Figure. S16 The blood biochemistry test and routine blood test results of samples collected from the mice inject with TCP (1 mmol/L in 1× PBS). Notes: the samples were collected and analyzed 7 days post different treatments ($n = 3$). Abbreviations: uric acid, UA; Creatinine, CREA; Albumin, ALB; white blood cell, WBC; Neutrophil, Neu; monocyte, Mon; lymphocyte, Lym; red blood cell, RBC; blood platelet, PLT; Mean corpuscular hemoglobin, MCH; hemoglobin, HGB. *P*-value >0.05 was defined as no significance (NS).

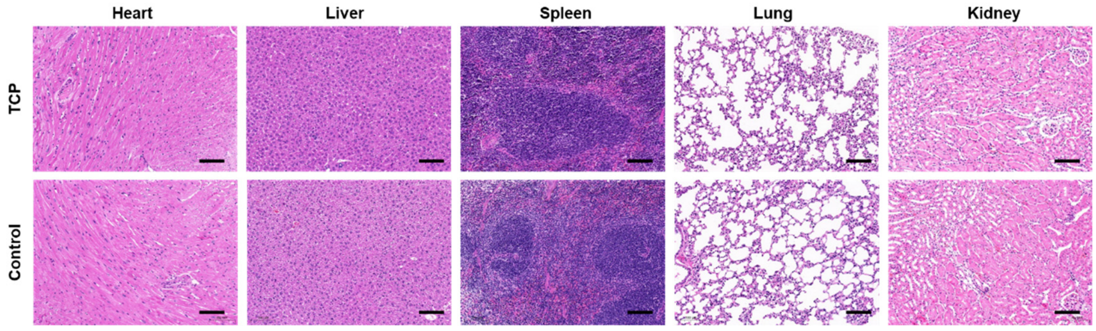

Figure. S17 H&E staining slices of major organs for the representative mice treated with TCP probe or normal saline control. scale bar: 100 μm.

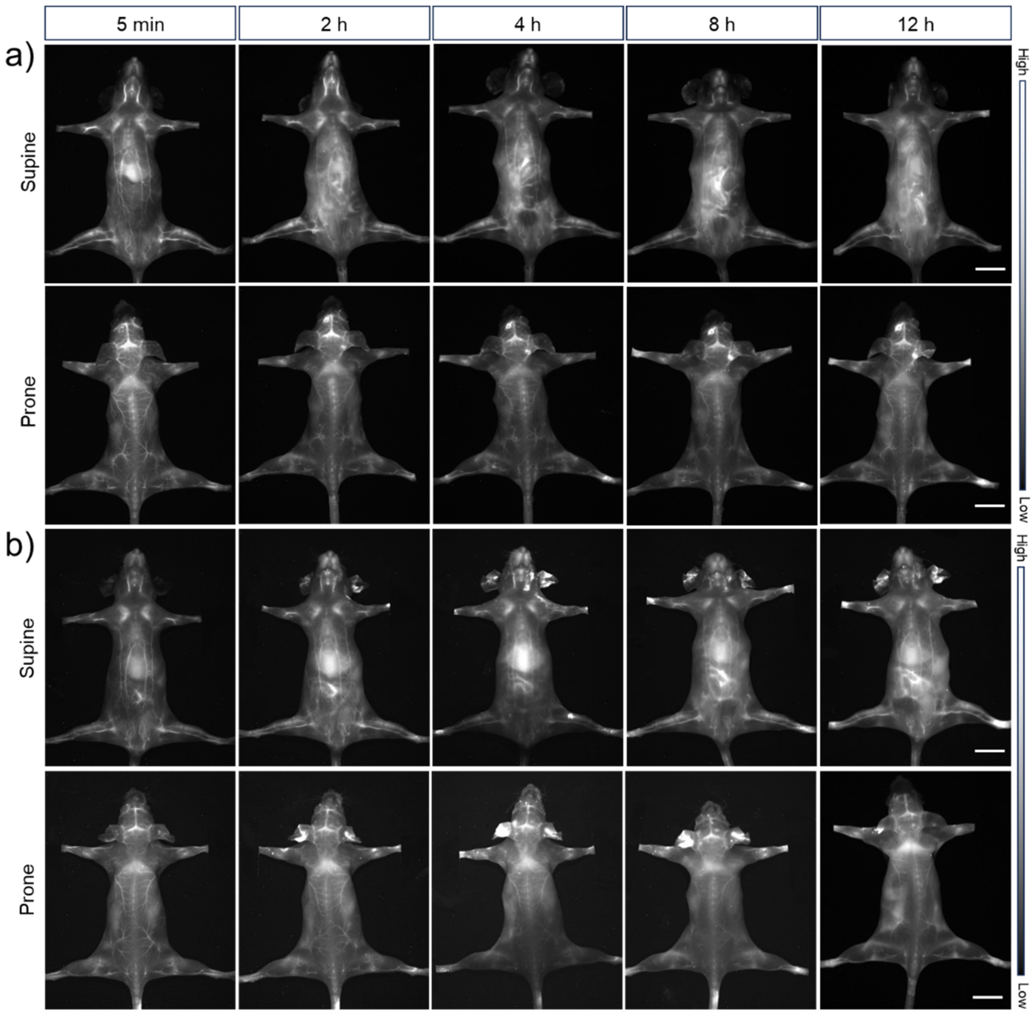

Figure. S18 The replicates of NIR-II fluorescence images of mice treated with TCP (laser: 808 nm, filter: LP1300 nm, exposure time: 500 ms, power: 30 mW/cm^2, scale bar: 1cm).

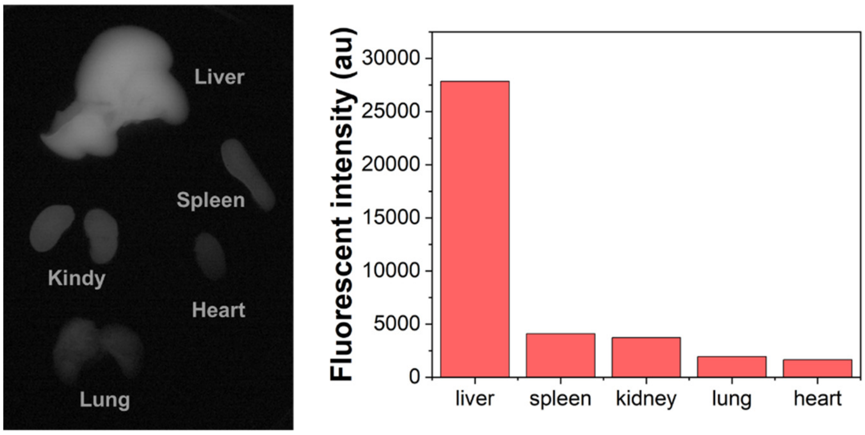

Figure. S19 The representative ex vivo NIR-II fluorescence imaging and quantitative analysis of different organs collected from mice treated with TCP (laser: 808 nm, filter: LP1300 nm, exposure time: 500 ms, power: 30 mW/cm^2).

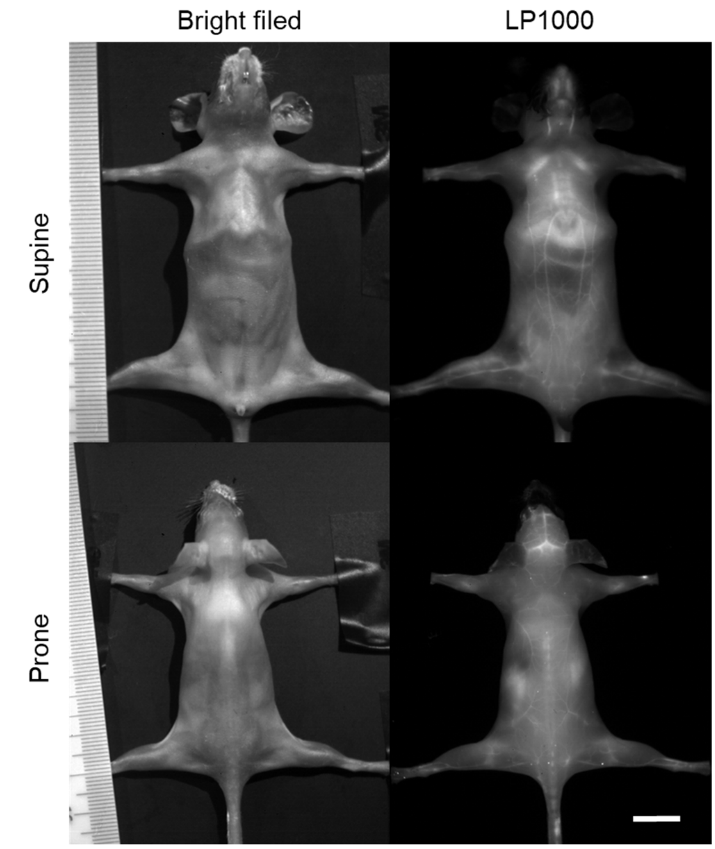

Figure. S20 The fluorescent angiography of the representative mouse in supine position and prone position after intravenous injection of TCP (200 μL, 1 mmol/L in 1× PBS, filter:LP1000 nm, exposure time: 10 ms, power: 30 mW/cm^2).
